# Is excessive online usage a function of medium or activity? An empirical pilot study

**DOI:** 10.1556/JBA.2.2013.016

**Published:** 2013-12-06

**Authors:** Mark D. Griffiths, Attila Szabo

**Affiliations:** ^1^Nottingham Trent University, Nottingham, United Kingdom; ^2^Eötvös Loránd University, Budapest, Hungary

**Keywords:** addiction, computer, dependence, habit, online, virtual environment

## Abstract

*Aims:* The purpose of the study was to seek a better insight into whether the online medium or the online activity was more important in relation to excessive online use. It is not clear whether those people who spend excessive amounts of time on the Internet are engaged *in general* Internet or whether excessive Internet use is linked to *specific activities*. *Methods:* Perceived changes in Internet use habits as function of hypothetical accessibility of favorite sites were investigated in young adults. University students (*n* = 130, mean age = 20.6 years) who had (on average) spent over 20 hours a week on the Internet for at least nine years completed a survey. The most favored online activities and expected quality of life without Internet access were also investigated. *Results:* Findings revealed that social networking was by far the most popular online activity, and that lack of access to their preferred online activities would drop by 65% (as measured by perceived Internet usage). Approximately one in six participants (16%) claimed they would not even switch on the computer if access to their favorite online activities were unavailable. In relation to a hypothetical question about the quality of life without Internet access, the responses were normally distributed (rather than skewed). *Conclusions:* These results show that time spent with Internet activity is not random and/or generalized, but appears more focused. Attraction or addiction on Internet to one or more *specific behavior(s)* may be a better way forward in the quest for better understanding excessive human behavior in the online environment.

## Introduction

The Global Internet User Survey based on over 10,000 respondents from 20 nations revealed 96% of the users accessed the Internet on a daily basis (The Internet Society, 2012). Excessive Internet usage, when accompanied by obsessive and/or compulsive symptoms (that may control the individual’s behavior), has been labeled as a modern psychological morbidity under the terminology ‘Internet addiction’ (Young, 1999a, 1999b). Today, this term is commonly adopted in the scholastic literature in spite of the fact that its ‘over generality’ has been recognized well over a decade (Griffiths, 2000a, 2000b, 2001). Indeed, the Internet is a virtual world with several unique environments and/or activities ranging from news and shopping to gambling and pornography. Theoretically, it is incorrect in most cases to label even a very severe Internet (environment)-based addiction as ‘Internet addiction’, because the latter fails to take into account the focus or the object of addiction (e.g., gambling, video gaming, social networking, shopping, sex, work, etc.).

It has been argued since the late 1990s that most of the people who spend excessive amounts of time on the Internet are not addicted to the medium itself, but exploit the Internet to fuel another and perhaps highly specific addiction (Griffiths, 2000a). Griffiths (2000a) noted that researchers should distinguish between *addictions on the Internet* and *addictions to the Internet*. He used the example of an addicted gambler and a video game addict who both choose to engage in their chosen behavior online, emphasizing that the Internet is just the environment where these people engage in addictive behaviors. Such individuals display addictions *on* the Internet rather than *to* it. However, there is also the observation that some behaviors engaged on the Internet (e.g., cybersex, cyber-stalking, etc.) may be behaviors that a person would only carry out on the Internet because the medium is anonymous, non-face-to-face, and disinhibiting (Griffiths, 2000b, 2001).

Despite the fact that the concept of ‘Internet addiction’ is arguably flawed, dismissing it entirely may be somewhat premature. For instance, in a clinical setting, ‘Internet addiction’ may be a useful diagnosis if the different possible objects of addiction all had comparable correlates and effective treatments. Similarly, substance use disorders have many different objects of addiction, but is generally accepted as a functional (i.e., more general) term. In addition to this, there are some activities that can only be carried out online (e.g., social networking) and therefore could be classed as a genuine ‘Internet addiction’ if problematic behavior arose (Kuss & Griffiths, 2011).

To better understand how and what people are specifically attracted to in the online environment, one needs to identify the preferred areas and assess expected (or even better, actual) changes if that area was removed or became unavailable. Such results may reveal whether changes in online behavior would occur. Two possible outcomes may be expected. The first is that removal of a preferred online environment will result in no change in regard to Internet usage patterns because the individuals would migrate to other available online environments. The second is that people would expect to spend less time on the Internet because their preferred online environment may no longer be accessed. The latter hypothesis is analogous to the theoretical (as well as practical) effects of smoking bans on the expected and actual prevalence of smoking (U.S. Department of Health & Human Services, 2006).

Therefore, in the current pilot study three questions were investigated: (i) What are the three most popular online activities among young Internet users?; (ii) What would be the expected changes (if any) in these online practices if the person’s favorite online activities could not be accessed?; and (iii) How would the perceived quality of life be affected if Internet access was terminated indefinitely for some reason? While the first and third questions were exploratory in nature, in context of the second question, the hypothesis that Internet access would decline if preferred online activities could not be accessed, was the specific interest of the current investigation. The current study was aimed at assessing attitudes regarding perceived Internet usage when preferred online activities are inaccessible, rather than actual Internet usage.

## Method

### Participants and procedure

From among 60 potential lecture groups at a large national university in Hungary, three groups from a research methods class were selected by random draw to participate in the study. All individuals selected by the draw agreed to participate (100% response rate) and no incentives to participate were given.

Participants in the three groups were asked to complete a short survey about their Internet use habits. This stratified, semi-random volunteer selection yielded 130 participants from the age group that, according to previous research, has the largest proportion of Internet users (Pew Research Center, 2012). All participants spoke the same language (Hungarian) and came from a similar social background. While the sample comprised more women, there were no differences in age and the reported weekly hours of Internet use, but males reported a longer history of Internet use than females (Table 1). Participants completed a survey-format questionnaire, containing the questions described below. The survey was performed in the morning, before lectures, in a classroom setting with prohibited interaction during the completion of the questionnaire. Local and international ethical guidelines were strictly followed in the course of the study (British Psychological Society, 2010).

### Measures

A single questionnaire was used comprising demographic questions, Internet use habits (history, estimated weekly hours), a specific question requesting participants to identify the three online activities they most used and percent time spent with each. The online activities that they could select from included: 1) *games and/or gambling,* 2) *general information and news* (including sports and politics), 3) *administration* (including banking, paying bills, etc.), 4) *music/culture,* 5) *videos and movies,* 6) *e-mail and chat,* 7) *meeting new friends (or love),* 8) *shopping,* 9) *adult content,* and 10) *social networking*. Further questions were: a) *If for technical reason(s), the most commonly used three online activities were not be accessible, would you still switch on your computer? If yes, how many hours would you spend on the Internet?;* b) *If the three most commonly used online activities that you engage in could no longer be accessed ever, how much time would you spend on the Internet (estimated percent)?;* c) *If Internet access was limited to only one activity, which one would you chose?;* and d) *If Internet access was no longer available, the quality of your life would be...* (rating scale 1 [worse] to 10 [better]).

**Table 1.** Means (and standard deviations) of participant characteristics (age, weekly Internet use, and Internet use history) classified by gender

	Men (*n* = 44)	Women (*n* = 86)
Age (years)	20.9 (2.7)	20.4(1.3)
Weekly Internet use (estimated hours)	21.3 (11.1)	21.1 (8.6)
History of Internet use (year)	11.1 (3.0)	9.4(2.4)^*^

^*^ Statistically significant difference between the mean (t(128) = 3.4, *p* < .001)

### Ethics

The study procedures were carried out in accordance with the Declaration of Helsinki. The Institutional Review Board of the Eötvös Loránd University (Faculty of Education and Psychology) approved the study. All subjects were informed about the study and all provided informed consent.

## Results

The three most popular Internet areas among the online users were: social networking (85%), e-mail and chat (69%), and videos and movies (35%). A majority (84%) of participants claimed they would still go online if their favorite three online activities were no longer available. However, in contrast to the reported average weekly use (21.13 hours; *SD* = 9.5), they expected to spend much less time online if their favorite online activities could not be accessed (7.43 hours; *SD* = 5.8), a difference that was significant (*t*(108) = –24.6, *p* < 01). However, 21 participants (16%) reported that they would not go online if their favorite online activities were unavailable.

**Figure 1.**
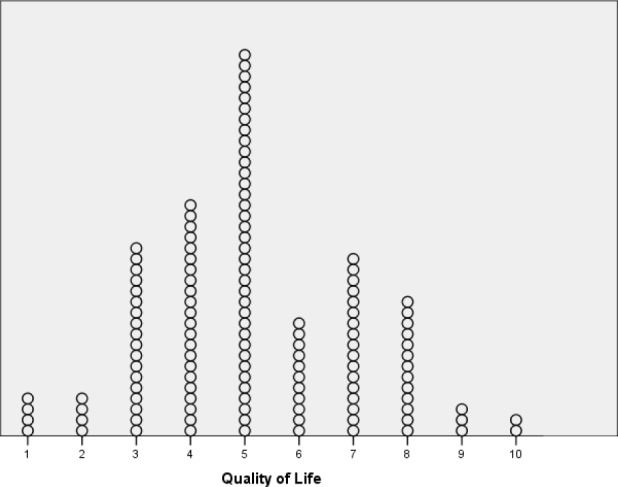
The distribution of the responses on a 10-point Likert scale to the question: *If Internet access was no longer available, the quality of your life would be*... (1 [= worse] to 10 [= better]; *n* = 130)

In response to the question: *If the three most commonly used online activities could no longer be accessed ever, how much time would you spend on the Internet (estimated percent)?,* the participants reported a 65% estimated decrease (*SD* = 17%). In response to the final question *If Internet access was no longer available, the quality of your life would be...* (rating scale 1 [worse] to 10 [better]), the participants’ responses were wide-ranging and exhibited a fairly normal distribution in their responses (mean = 5.15; *SD* = 1.9; median 5, range = 1–10, mode = 5; see Figure 1). There were no statistically significant gender differences in response to this question.

## Discussion

This pilot study examined perceived changes in Internet use habits as function of hypothetical accessibility of Internet use in a young adult sample who had (on average) spent over 20 hours a week on the Internet for at least nine years. The three most favored online environments were (i) social networking, (ii) e-mail and chat, and (iii) videos and movies. This appears to confirm research showing that social networking has become the most popular form of online activity, particularly among young people (Kuss & Griffiths, 2011). Results showed that lack of access to their preferred online activities, perceived Internet usage would drop by two-thirds (65%), and that approximately one in six participants (16%) claimed they would not even switch on the computer if their favorite online activities were unavailable. These results suggest that, in general, Internet users go online to engage in specific activities and that if they were prevented from doing so on their favorite activities, they would spend a lot less time online and would not necessarily engage in another online activity. These results also suggest that time spent online is not random and/or generalized, but appears (in general) to be specifically focused. There is, of course, an inherent bias in the results because if regular online users distribute their online time across many different activities and are then prevented from engaging in their favorite ones, they would naturally report that they would spend less time participating in online activities overall. However, even with the expected bias, the results therefore support the claim that most people go online to engage in specific online activities rather than for generalized Internet use.

In relation to a hypothetical question about the quality of life without Internet, the responses were normally distributed (rather than skewed). This suggests that for some people, the engaging in online activities is fundamental for psychological wellbeing in their lives and for others it is not. It also suggests that some people (even those who are presently regular and arguably heavy Internet users) feel they could happily live without the Internet. However, some heavy Internet users may feel that their Internet use is currently causing problems in their lives. In these cases, not having access to the Internet might be perceived by such people as improving their quality of life (i.e., it is currently fundamental in their lives but their lives might be better if Internet access was unavailable). Additionally, the data as a whole should be treated with caution given that these individuals are unlikely to have ever had a long period without Internet access. Saying how they think they would feel may be very different from how they would actually feel if such a situation were ever to arise.

The results also suggest that attraction and/or addiction on the Internet to one or more *specific behavior(s)* may be a correct conceptualization in the quest for better understanding and explaining human behavior in online environments. Furthermore, it provides some empirical evidence that research into Internet addiction should perhaps focus on specific online activities (e.g., social networking, online gaming, online gambling, etc.) rather than generalized Internet use. This has been argued for by some researchers for well over a decade (Griffiths, 2000a), and provides a good rationale and face validity as to why separate screening instruments for such specific Internet activities such as online gaming (King, Haagsma, Delfabbro, Gradisar & Griffiths, 2013) and social networking (Andreassen, Torsheim, Brunborg & Pallesen, 2012) have been developed.

As far as the authors are aware, this is the first ever study to investigate what individuals would do if they were prevented from gaining access to their preferred online activities, and is therefore of existential value. Given that individuals in this study are never likely to experience long periods without Internet access, questions asking what they thought they might do in such scenarios appeared to be the most appropriate methodological approach (although as highlighted above, this is not without its problems). However, there are clearly a number of other limitations to the pilot study including a small sample size, the reliance on self-report and all the problems that are associated with such a methodology (e.g., social desirability biases, recall biases, etc.), and the fact that participants had to hypothesize what they would do if there was no Internet access (when in reality such a scenario is never likely to happen on anything but a short-term basis).

Some of the online activities listed (e.g., reading general information and news, or adult content) may have meant different things to different people. Furthermore, there may be some confounding variables as some of the online activities may serve essential purposes. For instance, because the sample comprised students, they may be required to use the Internet as part of their academic studies (e.g., research, e-mail, etc.). Another potential issue relates to how the participants accessed the Internet. No information was collected on whether their favorite online activities were accessed by computer terminal, laptop, tablets, and/or mobile phones. This may have impacted on the findings.

Therefore, if future research studies were to be carried out in this area, they should be done with much larger sample sizes to confirm or negate the findings presented here. Future research might be better served by recruiting people who are addicted to the Internet rather than looking at trends within the general population. To demonstrate differences between people addicted *to* the Internet *versus* those with addictions *on* the Internet, studies would need to follow-up with people who reported that their Internet usage would stay constant even if they were prevented from engaging in their favorite activities. It would be also interesting to test the hypothesis empirically. This could potentially be done by using programs that ban access to certain websites and then tracking how long time people spend on the Internet. Such a study might show that a small population of people spend just as much time online by visiting different websites and/or engaging in different online activities.
